# ‘Finishing the race’ – a cohort study of weight and blood glucose change among the first 36,000 patients in a large-scale diabetes prevention programme

**DOI:** 10.1186/s12966-022-01249-5

**Published:** 2022-01-26

**Authors:** Antonia M. Marsden, Peter Bower, Elizabeth Howarth, Claudia Soiland-Reyes, Matt Sutton, Sarah Cotterill

**Affiliations:** 1 Centre for Biostatistics, School of Health Sciences, University of Manchester, Oxford Road, Manchester, M16 0BY UK; 2 Centre for Primary Care and Health Services Research, NIHR ARC Greater Manchester, School of Health Sciences, University of Manchester, Manchester, UK; 3 Research & Innovation, Northern Care Alliance NHS Group, Manchester, UK

**Keywords:** Diabetes prevention, Weight loss, HbA1c reduction, Public health, Programme evaluation

## Abstract

**Background:**

The NHS Diabetes Prevention Programme for England, “Healthier You”, encourages behaviour change regarding healthy eating and physical exercise among people identified to be at high risk of developing type 2 diabetes. The aim of this research was to examine change, and factors associated with change, in measures of HbA1c and weight in participants and completers of the programme between 2016 and 2019.

**Methods:**

Participant-level data collected by programme service providers on referrals prior to March 2018 was analysed. Changes from baseline to both 6 months and completion in HbA1c and weight were examined using mixed effects linear regression, adjusting for patient characteristics, service provider and site.

**Results:**

Completers had average improvements in HbA1c of 2.1 mmol/mol [95% CI: − 2.2, − 2.0] (0.19% [95% CI: − 0.20, − 0.18]) and reductions of 3.6 kg [95% CI: − 3.6, − 3.5] in weight, in absolute terms. Variation across the four providers was observed at both time points: two providers had significantly smaller average reductions in HbA1c and one provider had a significantly smaller average reduction in weight compared to the other providers. At both time points, ex- or current smokers had smaller reductions in HbA1c than non-smokers and those from minority ethnic groups lost less weight than White participants. For both outcomes, associations with other factors were small or null and variation across sites remained after adjustment for provider and case mix.

**Conclusions:**

Participants who completed the programme, on average, experienced improvements in weight and HbA1c. There was substantial variation in HbA1c change and smaller variation in weight loss between providers and across different sites. Aside from an association between HbA1c change and smoking, and between weight loss and ethnicity, results were broadly similar regardless of patient characteristics.

**Supplementary Information:**

The online version contains supplementary material available at 10.1186/s12966-022-01249-5.

## Introduction

Both the incidence and prevalence of type 2 diabetes is increasing globally and prevention has become a major international public health objective [1–3]. Whilst many risk factors of diabetes are unmodifiable (genetics, age, ethnicity), obesity is known to elevate the risk [1, 4]. Prevention of diabetes is therefore centred on changes in behaviour such as eating a healthier diet and increasing physical activity [5–7].

Many countries have initiated diabetes prevention programmes to encourage positive behaviour change in those at high risk of developing type 2 diabetes. Research in both trial and real-world settings have suggested these programmes can be effective. Systematic reviews assessing the effectiveness of diabetes prevention programmes worldwide have found these programmes are effective in reducing the incidence of type 2 diabetes and result in weight loss [8–11].

The NHS Diabetes Prevention Programme for England, “Healthier You”, encourages behaviour change regarding healthy eating and physical exercise among people identified to be at high risk of developing type 2 diabetes. The programme was developed by an expert group and incorporates mechanisms shown to be clinically effective at influencing behaviour. The programme was rolled out across England in three annual waves between 2016 and 2018. The target population was adults aged 18 and over with non-diabetic hyperglycaemia (NDH), defined as glycated haemoglobin (HbA1c) of 42–47 mmol/mol (6·0–6·4%) or fasting plasma glucose level (FPG) of 5·5–6·9 mmol/l. Participants were referred to the programme through their GP via one of two main routes: i) referral by a primary care professional following a consultation, or ii) self-referral following receipt of a letter from their GP, informing them of their high risk of type 2 diabetes (based on their medical records) and encouraging them to participate [12]. Delivery was provided by four independent service providers commissioned locally in each site – generally defined at Sustainability and Transformation Partnership (STP) level [13]. Once referred, participants were offered an individual initial assessment, followed by contact of at least 16 h over nine to twelve months, involving regular group education and exercise sessions [14].

We aimed to examine changes in measures of HbA1c and weight in participants and completers of the Healthier You Diabetes Prevention Programme between 2016 and 2019, and to report variation in outcomes by patient characteristics, service provider and geographical area.

The service delivery team has previously published findings based on an earlier version of the data [15]. Here, we independently both confirm the robustness of previous findings and extend this previous analysis, providing new insights. Compared to Valabhji et al., we measure change over different, more consistent periods and use a different measure of baseline weight, which we believe is more appropriate. Uniquely, we report associations between changes in outcomes with disability, employment status, smoking and well-being scores. Our modelling considers plausible causal relationships and employs multiple models to achieve appropriate adjustment for reported associations. Lastly, we explore variation by site in more detail.

## Research design and methods

### Study population

This observational cohort study used patient-level data collected by the NHS Diabetes Prevention Programme (NHS DPP) service providers, recording participation and outcomes for all referrals received by them since April 2016 [16]. Data were extracted in September 2019. We analysed referrals received prior to March 2018, allowing eighteen months from referral to completion in order to exclude those potentially still participating at the time of the extract, although participants who took longer than eighteen months may still have been participating.

### Participant characteristics

Demographic data collected by providers included sex, age, ethnicity, 2015 index of multiple deprivation (IMD) score for their small-area of residence grouped using quintiles, employment status, disability and smoking. Well-being measures were also collected, either using the Warwick Edinburgh Mental Wellbeing Scale (WEMWBS) or EQ-5D depending on date of the initial assessment [17, 18].

HbA1c or FPG was recorded at referral (measured in primary care) and was only measured again by providers at initial assessment if it was missing or more than 3 months old. Blood measures were repeated at 6 months after the first intervention session and at completion for those still attending. Since HbA1c measures at referral were obtained using venous techniques while those recorded subsequently by providers used point-of-care tests, the initial assessment measure was used as the baseline measure for comparability with the recorded follow-up measures [19]. Multiple imputation was used to impute initial assessment HbA1c measures where these where not measured (30.9% of the sample).

Weight was measured at initial assessment. Subsequent weight measurements were recorded at each intervention session attended; for comparability with the analysis of HbA1c, a 6 month weight measure was identified as that recorded closest to and within 31 days of the 6 month HbA1c measure where the latter date was available, or otherwise was counted as missing. The final weight measure at completion was defined as that recorded closest to and within 31 days of the final HbA1c measure where available, or otherwise that recorded at the final session where this was attended, or otherwise classed as missing.

Service features, provider and session times were recorded. ‘Out-of-hours’ provision describes any session starting outside the hours of 09:00 to 17:00. Commissioning site was described by both Clinical Commissioning Group (CCG), local primary care led statutory bodies which commission health services, and, at a higher level, STP, which led on planning for the long term needs of areas at that time. In general, each STP commissioned a single provider but CCGs managed the local implementation of referrals from primary care [13].

The data collection methods are described fully elsewhere [20].

### Outcomes

We examined change in HbA1c and weight from (i) initial assessment to 6 months and (ii) initial assessment to completion. We examined variation in each outcome associated with patient and service features, and across sites (both STP and CCG). Analyses at each time point were restricted to those retained to that time (*n* = 36,614 at 6 months, *n* = 22,697 at completion), since outcomes for those lost to follow-up were thought likely to be missing not at random. Participants with any 6 month or final measure recorded or a period of attendance of at least 180 days from the first attended intervention session (even if they did not provide 6 month or final outcome data) were classed as retained to 6 months. Those with attendance at the final intervention session or any final measure recorded plus at least 60% attendance were classed as having completed the programme. Based on the date of the final HbA1c measure, the median time between initial assessment and completion was 345 days (IQR: 294, 436).

### Statistical analysis

Multiple imputation was used to reduce bias due to missingness of outcomes and demographic data of up to 44% (Table S5). Planned analyses, and therefore imputation, were restricted to those retained to at least 6 months. See Additional file 1 for further details of the imputation process.

Raw changes were calculated between baseline and both 6 months and completion. Changes were re-assessed having applied multiple imputation for those who did not have an observed outcome at 6 months or completion but who were still attending the programme at this time. Linear regression models were applied to imputed data to estimate adjusted associations of sex, age, deprivation, disability, occupation, smoking status, well-being, provider, referral source and out-of-hours provision with each outcome. Additionally, associations of HbA1c at baseline with changes in weight and weight at baseline with changes in HbA1c were examined. The assumptions of linear relationships between continuous covariates and outcomes were found to be acceptable using exploratory plots. Causal relationships were considered in order to adjust for measured confounders while avoiding adjustment for intermediaries or common descendants [21, 22]. For example, in assessing the association between ethnicity and outcomes, we did not adjust for deprivation as this is a plausible mediator on the causal pathway [23].

In some cases, site was identified as a potential intermediary between the characteristic and some outcomes. For example, where an individual lives may be on the causal pathway between age/ethnicity and the outcomes. In these cases, fixed effects linear regression was used with robust estimation to allow for clustering by CCG, providing estimates of marginal associations across sites. Otherwise, mixed effects models with nested independent random intercept terms for CCG within STP were used to allow for clustering. Both CCG and STP were included to explore potential clustering at both levels. Variation across sites was investigated using coverage intervals for the predicted mean of each outcome for individuals with typical combinations of characteristics (see Additional file 1 for details).

## Results

### Patient characteristics

Summaries of patient characteristics among all who started the programme by attending an initial assessment, termed ‘attenders’, and among subgroups retained to 6 months and completion are shown in Table 1. A more detailed summary, including a summary by provider, is given in Table S1. The overall proportion of women was 54% and the median age was 66 (IQR 58, 75) years with 59% aged between 60 and 79 years. Proportions were close to 20% in each of the five deprivation strata but with slightly higher proportions from more deprived areas compared with less deprived areas. Proportions 76, 13, 7 and 4% reported White, Asian, Black and other ethnicities respectively. The most common employment status was retired (58%) and 17% reported a disability. There was considerable variation across the four service providers with respect to deprivation, less so for other characteristics. Those retained to 6 months and completion tended to be slightly older and less deprived on average, more likely to be White and more likely to be retired.

**Table 1:** Demographic summary and service characteristics for attenders, those retained to 6 months and those who completed

	Attenders	Retained to 6 months	Completed
N	99,131	36,614	22,697
Male sex (%)	44.5	45.1	45.2
Age			
Median (IQR)	66 (17)	68 (14)	69 (12)
< 40	3.6	1.6	1.1
40–49	9.2	5.5	4.3
50–59	19.5	16.0	14.3
60–69	30.1	34.2	35.2
70–79	28.5	34.1	36.4
80+	9.1	8.6	8.8
Deprivation (%)			
1 (most deprived)	19.2	13.5	12.2
2	19.8	17.6	18.0
3	20.1	21.2	22.3
4	20.0	22.5	22.9
5 (least deprived)	20.9	25.2	24.6
Ethnicity^a^ (%)			
White	76.1	81.5	83.9
Asian	12.9	9.2	7.8
Black	7.2	6.0	5.3
Mixed or other	3.8	3.4	3.0
Employment (%)			
Employed	31.8	25.8	23.5
Retired	57.7	66.5	69.8
Other	10.5	7.7	6.8
Disability (%)	17.4	15.5	14.9
Smoking (%)			
Smoker	8.2	5.0	4.6
Ex-smoker	2.4	2.1	1.9
Non-smoker	89.4	92.9	93.6
WEMWBS score			
Median (IQR)	54 (13)	54 (12)	55 (12)
EQ-5D VAS			
Median (IQR)	80 (25)	80 (20)	80 (20)
Referral source (%)			
Consultation (GP/ health check)	63.8	65.3	60.2
Letter (self-referral after advice)	36.2	34.7	39.8
Out-of-hours provision (%)			
Some	15.5	18.9	15.3

^a^ ‘Asian’ comprises those reporting Indian, Pakistani, Bangladeshi, Chinese or ‘other Asian’ ethnicity; ‘Black’ comprises those reporting Caribbean, African or ‘other Black’ ethnicity; ‘Mixed’ comprises people with a Mixed ethnic background and ‘Other’ comprises those reporting any other ethnicity

### Service features

Descriptive summaries of service features are also given in Table 1. The majority (64%) of attenders were referred via the consultation route, with 36% via the letter route. It is unknown how many candidates declined a referral at consultation or failed to self-refer after receiving an invitation by letter. Of the attenders, 16% scheduled at least one session that was out of hours. There was considerable heterogeneity across providers in referral numbers, referral source and out-of-hours provision.

### Descriptive summaries of outcomes

Descriptive summaries of observed HbA1c and weight measures at initial assessment, 6 months and completion are available in the Additional file 1 (Table S2). Median HbA1c at initial assessment was 41 mmol/mol [IQR 39, 43] (5.9% [IQR 5.7, 6.2]); median FPG was 6.0 mmol/l [IQR 5.7, 6.3] but numbers with FPG reported at any subsequent time point were so small that no further analysis of FPG was attempted. Over half of all attenders with HbA1c or FPG recorded at initial assessment had measures in the normal range compared with just 1% of referral measures (venous tests) among the same group. This change may be attributable to genuine reductions in HbA1c after participants were informed that they were at risk of developing type 2 diabetes, or due to measurement error as previous work has suggested point-of-care tests tend to give lower readings than venous methods [19].

Median weight at initial assessment was 82 kg [IQR 70, 94] with 37% classed as overweight and 46% as obese. Similar baseline distributions for all measures were seen among those retained to 6 months and completion.

Descriptive summaries of observed changes in HbA1c and weight measures at 6 months and completion are shown in Table 2. Mean change in HbA1c was − 1.7 mmol/mol [SD 4.3] (− 0.16% [SD 0.39]) at 6 months, − 2.3 mmol/mol [SD 4.3] (− 0.21% [SD 0.39]) at completion. Of those with measures in the NDH range at initial assessment, 60% at 6 months and 68% at completion had measures in the normal range. Mean change in weight was − 3.2 kg (SD 4.4) at 6 months and − 3.6 kg (SD 4.8) at completion, corresponding to mean percentage weight changes of − 3.8% and − 4.2% respectively. Summaries of observed changes in HbA1c and weight stratified by patient and service features are shown in Table S3.

**Table 2:** Unadjusted change in health measures at 6 months and completion

	Retained to 6 m (n = 36,607)	Completed (n = 22,697)
	IA to 6 m	IA to completion
**Change in HbA1c (mmol/mol)**^a^		
n	20,682	15,017
mean (SD)	−1.7 (4.3)	− 2.3 (4.3)
**Change in HbA1c (%)**		
n	20,682	15,017
mean (SD)	−0.16 (0.39)	−0.21 (0.39)
**Change in HbA1c category**^b^		
**Normal at IA**		
Normal at follow up	8896 (85.4)	6324 (87.4)
NDH at follow up	1456 (14.0)	859 (11.9)
T2D at follow up	63 (0.6)	49 (0.7)
**NDH at IA**		
Normal at follow up	5782 (60.1)	5002 (68.2)
NDH at follow up	3575 (37.2)	2196 (29.9)
T2D at follow up	257 (2.7)	142 (1.9)
**T2D at IA**		
Normal at follow up	265 (39.5)	233 (51.3)
NDH at follow up	260 (38.8)	165 (36.3)
T2D at follow up	146 (21.8)	56 (12.3)
**Change in weight (kg)**^c^		
n	24,949	19,794
mean (SD)	−3.2 (4.4)	−3.6 (4.8)
**Percentage change in weight**		
n	24,949	19,794
mean (SD)	−3.8 (5.0)	−4.2 (5.5)
**Change in BMI (kg/m**^**2**^**)**		
n	24,801	19,667
mean (SD)	−1.2 (1.6)	−1.3 (1.7)

Abbreviations: *IA* initial assessment, *NDH* non-diabetic hyperglycaemia, *T2D* type 2 diabetes

^a^ These are descriptive summaries of those with a measure recorded at both initial assessment and the follow-up point. Many participants were not due to have HbA1c measured at initial assessment if their referral measure was less than 3 months old. All retained patients were included in the adjusted analysis after multiple imputation of missing initial assessment values

^b^ ‘Normal’ range: HbA1c < 42 mmol/mol or FPG < 5.5 mmol/l; ‘NDH’ range: HbA1c 42-47 mmol/mol or FPG 5.5–6.9 mmol/l inclusive; ‘T2DM’ range: HbA1c > 47 mmol/mol or FPG > 6.9 mmol/l

^c^ Six-month [final] weight is missing where no dated weight was recorded within 31 days of the six-month [final] HbA1c measure, or where no date is recorded for a six-month [final] HbA1c measure [and where no final-session weight was recorded]

### Change in HbA1c

**Fig. 1:**
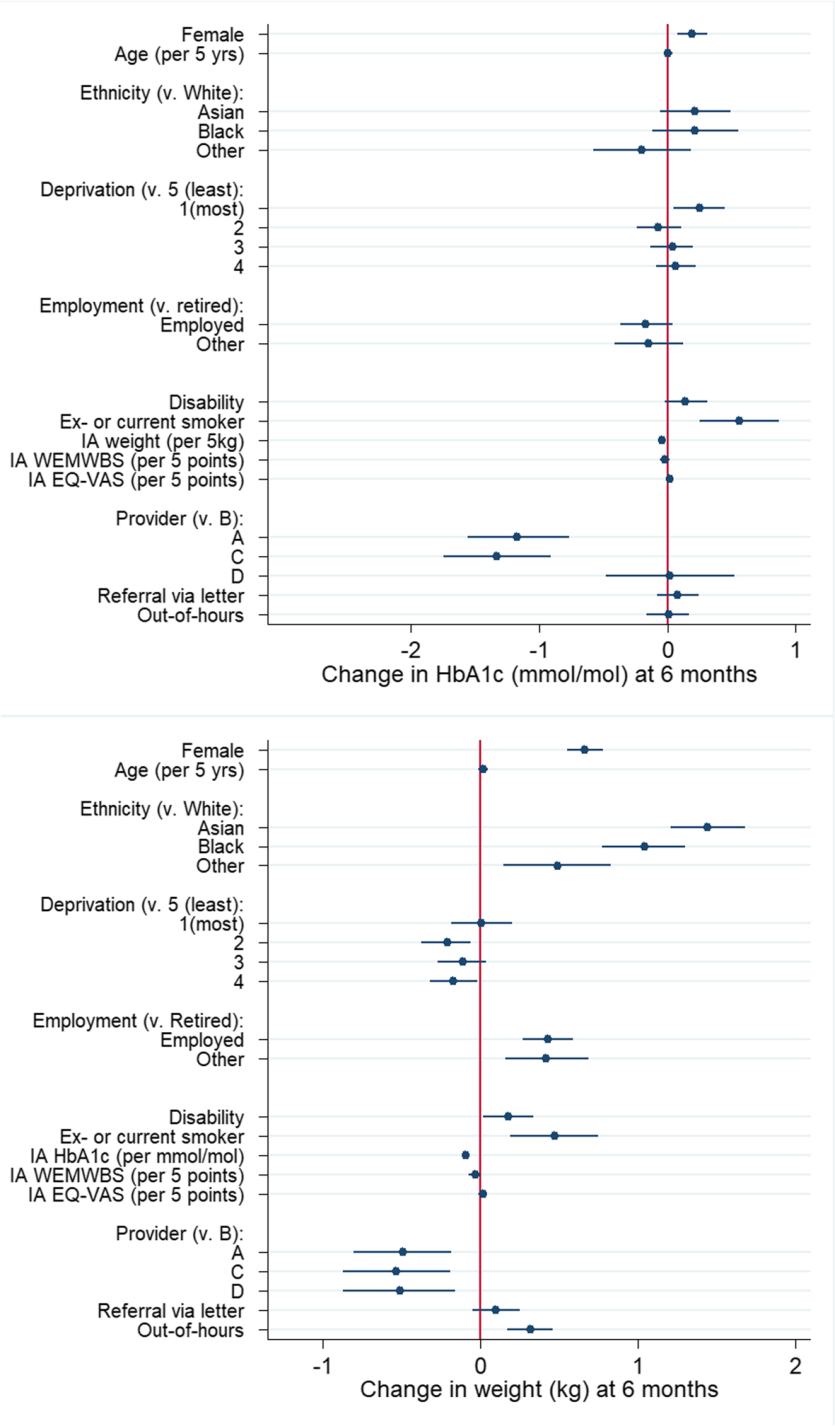
Association between patient and service characteristics and absolute change in HbA1c and weight at 6 months, measured relative to the reference category (for categorical variables)

Estimated associations between patient and service features and change in weight and HbA1c at 6 months are shown in Table 3 and Fig. 1. Associations with change in weight and HbA1c at completion are available in the Additional file 1 (Table S4). A negative change indicates a reduction in weight or HbA1c, hence a negative coefficient suggests a larger reduction and a positive coefficient suggests a smaller reduction, in comparison to the reference category.

**Table 3:** Associations of patient and service characteristics with change in HbA1c and weight at 6 months

	Change in HbA1c IA-6 m			Change in weight (kg) IA-6 m		
	Coef	SE	95% CI	Coef	SE	95% CI
**Sex**^a^						
Female	0.19	0.06	0.08, 0.31	0.66	0.06	0.55, 0.78
Male	0 (ref)			0 (ref)		
**Age**^a^ **per 5 years**	0.002	0.02	−0.03, 0.04	0.02	0.02	− 0.01, 0.05
**Ethnicity**^ab^						
Asian	0.21	0.14	−0.06, 0.49	1.44	0.12	1.21, 1.68
Black	0.21	0.17	−0.12, 0.55	1.04	0.13	0.77, 1.30
Other	−0.20	0.19	−0.58, 0.18	0.49	0.17	0.15, 0.83
White	0 (ref)			0 (ref)		
**Deprivation**^cd^						
1 (most deprived)	0.25	0.10	0.05, 0.45	0.01	0.10	−0.18, 0.20
2	−0.07	0.09	−0.24, 0.11	− 0.21	0.08	− 0.37, − 0.06
3	0.04	0.09	−0.13, 0.20	− 0.11	0.08	− 0.27, 0.04
4	0.06	0.08	−0.09, 0.22	−0.17	0.08	−0.32, − 0.02
5 (least deprived)	0 (ref)			0 (ref)		
**Disability**^cd^						
Yes	0.14	0.08	−0.02, 0.31	0.18	0.08	0.02, 0.34
No	0 (ref)			0 (ref)		
**Employment**^cd^						
Employed	−0.17	0.10	−0.37, 0.04	0.43	0.08	0.27,0.59
Retired	0 (ref)			0 (ref)		
Other	− 0.15	0.13	−0.41, 0.12	0.42	0.13	0.16, 0.69
**Smoking**^cd^						
Ex- or current smoker	0.56	0.16	0.25, 0.87	0.47	0.14	0.19, 0.75
Non-smoker	0 (ref)			0 (ref)		
**IA weight (per 5 kg)**^de^	−0.04	0.01	−0.06, − 0.02	n/a	n/a	n/a
**IA HbA1c (per mmol/mol)**^de^	n/a	n/a	n/a	−0.09	0.01	−0.10, − 0.07
**IA WEMWBS (per 5 points)** ^df^	− 0.02	0.02	− 0.06, 0.02	− 0.03	0.02	− 0.07, 0.01
**IA EQVAS (per 5 points)** ^df^	0.02	0.01	−0.01,0.05	0.02	0.01	−0.01, 0.04
**Provider**^dg^						
A	−1.17	0.20	−1.56, − 0.77	−0.49	0.16	−0.80, − 0.18
B	0 (ref)			0 (ref)		
C	−1.33	0.21	−1.75, − 0.91	−0.53	0.17	−0.87, − 0.19
D	0.02	0.25	−0.48, 0.52	−0.51	0.18	− 0.87, − 0.16
**Referral source**^dg^						
Letter	0.08	0.08	−0.08, 0.24	0.10	0.08	−0.05, 0.25
Consultation	0 (ref)			0 (ref)		
**Out-of-hours provision**^dg^						
Some	0.01	0.08	−0.16, 0.17	0.32	0.08	0.17, 0.46
None	0 (ref)			0 (ref)		
${\hat{\boldsymbol{\sigma}}}_{\boldsymbol{STP}}^{\mathbf{2}}+{\hat{\boldsymbol{\sigma}}}_{\boldsymbol{CCG}}^{\mathbf{2}}$	0.40			0.14		
**ICC (CCG level)**	0.02			0.01		

For categorical variables, ‘coef’ represents the adjusted difference in mean change in relation to the reference category. For numerical variables, ‘coef’ represents the adjusted difference in mean change between consecutive values. Change is calculated as the 6 month value minus the baseline value

^a^ Associations of sex, age and ethnicity with each outcome were mutually adjusted and marginal over site (no random terms)

^b^ ‘Asian’ comprises those reporting Indian, Pakistani, Bangladeshi, Chinese or ‘other Asian’ ethnicity; ‘Black’ comprises those reporting Caribbean, African or ‘other Black’ ethnicity; ‘Mixed’ comprises people with a Mixed ethnic background and ‘Other’ comprises those reporting any other ethnicity

^c^ Associations of disability, employment, deprivation and smoking with each outcome were mutually adjusted and adjusted for sex, age and ethnicity

^d^ Random intercept terms for STP & CCG included; associations are conditional, representing the estimated individual-level mean association after random variation between sites has been accounted for

^e^ Association of initial weight with change in HbA1c, and of initial HbA1c with change in weight, was adjusted for sex, age, ethnicity, employment, disability, deprivation and smoking

^f^ Association of WEMWBS score or EQVAS score with change in HbA1c [weight] was adjusted for sex, age, ethnicity, employment, disability, deprivation, smoking and initial weight [HbA1c]

^g^ Associations of service features with change in HbA1c [weight] were mutually adjusted and adjusted for sex, age, ethnicity, deprivation, smoking, initial weight [HbA1c] and service maturity

After multiple imputation, the estimated mean change in HbA1c was − 1.6 mmol/mol [95% CI -1.7, − 1.6] (− 0.15% [95% CI: − 0.16, − 0.15]) at 6 months and − 2.1 mmol/mol [95% CI -2.2, − 2.0] (− 0.19% [95% CI: − 0.20, − 0.18]) at completion. The decrease was smaller for ex- or current smokers compared with non-smokers at 6 months (0.56 mmol/mol [0.25, 0.87]) indicating that ex/current smokers had a reduction that was 0.56 mmol/mol less than non-smokers, on average (Table 3). The decrease was slightly smaller for women than for men (0.19 mmol/mol [0.08, 0.31]), and higher weight at initial assessment was associated with slightly larger reduction in HbA1c (− 0.04 mmol/mol [− 0.07, − 0.02] per 5 kg). Compared to the least deprived quintile, individuals in the most deprived quintile had a slightly smaller reduction in HbA1c at 6 months (0.25 mmol/mol [0.05, 0.45]) but differences with other quintiles were not observed. Age, ethnicity, disability, employment and well-being at baseline were not associated with change in HbA1c after adjustment for potential confounders. After adjustment for case mix, two providers saw substantially larger reductions on average (− 1.33 mmol/mol [− 1.75, − 0.91] and − 1.17 mmol/mol [− 1.56, − 0.77]) at 6 months compared with the reference provider (provider B). Neither referral source nor out-of-hours provision were associated with change in HbA1c.

Associations with change in HbA1c from initial assessment to completion were similar to those seen at 6 months (Table S4), aside from differences in change between the most and least deprived quintiles were no longer observed and there was some evidence that Black participants had a smaller reduction in comparison to White participants (0.48 mmol/mol [0.03, 0.93]).

Calculation of coverage intervals showed that, after adjustment for case mix and service features, the predicted mean change in HbA1c lay between − 3.3 and − 0.8 mmol/mol (− 0.30 and − 0.07%) at 6 months and − 3.9 and − 1.2 mmol/mol (− 0.36 and − 0.11%) at completion across the middle 95% of sites (ICC 0.02 and 0.03 respectively at CCG level) for individuals with typical characteristics (see Additional file 1 for details). The width of these intervals illustrate considerable residual variation across sites regarding change in HbA1c.

### Change in weight

After multiple imputation, the estimated mean change in weight was − 3.2 kg [95% CI: − 3.3, − 3.1] at 6 months and − 3.6 kg [95% CI: − 3.6, − 3.5] at completion. At 6 months, those from minority ethnic groups lost less weight than White participants (1.44 kg [1.21, 1.68], 1.04 kg [77, 1.30] and 0.49 kg [0.15, 0.83] for those from Asian, Black and other groups respectively). Smaller associations were observed with sex, employment and smoking status: women lost slightly less weight than men (0.66 kg [0.55, 0.78]), non-retired participants lost less weight than retirees (0.43 kg [0.27, 0.59] and 0.42 kg [0.16, 0.99] for employed and other non-retired participants respectively) and ex- and current smokers lost less weight than non-smokers (0.47 kg [0.19, 0.75]) (Table 3). Slight differences were seen across deprivation groups but with no clear trend and disabled participants lost slightly less weight than those with no disability. Higher HbA1c at initial assessment was associated with slightly greater weight loss (− 0.09 kg [− 0.10, − 0.07] per mmol/mol). Age and well-being score at initial assessment were not associated with change in weight after adjustment for potential confounders.

After adjustment for case mix, three providers saw similar levels of weight loss, which were, on average, − 0.49 kg [− 0.80, − 0.18], − 0.53 kg [− 0.87, − 0.19] and − 0.51 kg [− 0.87, − 0.16] larger than provider B. Those with some out-of-hours provision lost slightly less weight than those with none. Associations with change in weight from initial assessment to completion were similar to those seen at 6 months (Table S4).

Calculation of coverage intervals showed that, after adjustment for case mix and service features, the predicted mean change in weight lay between − 4.4 kg and − 2.9 kg at 6 months and − 4.8 kg and − 3.2 kg at completion across the middle 95% of sites (ICC 0.01 at CCG level at each time-point), illustrating moderate residual variation across sites.

## Discussion

### Summary of findings

Both HbA1c and weight decreased on average among those retained to at least 6 months across all patient subgroups, providers and sites.

The reductions in HbA1c were similar across patient subgroups. Ex- or current smokers had smaller reductions in HbA1c than non-smokers at both time points, and Black participants had smaller reductions than White participants at completion, but these were small between-group differences of 0.6 mmol/mol or less. Provider variation was found to be a more important factor, with differences of up to 1.3 mmol/mol in mean reduction at 6 months between providers after adjusting for case mix. Importantly, considerable variation across sites was seen for HbA1c reduction after accounting for case mix and provider, which may reflect small differences in local implementation of the diabetes prevention service or differences in the configuration of other local health services.

Reductions in weight were largest among White participants, with smaller mean reductions among all minority ethnic groups (by up to 1.4 kg among Asian participants) at 6 months. Female sex, being non-retired, ex- or current smoking and disability were also associated with a smaller reduction in weight, but to a lesser degree. Aside from ethnicity, estimated differences between groups for all other patient characteristics were 0.7 kg or less. Less variation across providers was seen for weight change than for HbA1c after adjusting for case mix, with similar means among three of the four providers and a smaller mean reduction by approximately 0.5 kg at 6 months for the fourth. Variation in weight across sites was lower than for HbA1c, but still present. Those participants with some out-of-hours provision lost slightly less weight on average than those with none, but were less likely to be retired and more likely to be from a minority ethnic group, both associated with reduced weight loss, so this result may reflect an interaction of these associations.

Although effect sizes were small, a higher baseline HbA1c was associated with a larger reduction in weight, and a higher baseline weight was associated with a larger reduction in HbA1c, suggesting better improvements for those at highest risk.

### Comparison with the literature

Using the same dataset, Valabhji et al. analysed (i) change from baseline to the last available measure, reporting a mean HbA1c change of − 1.3 mmol/mol (− 1.3, − 1.2) and a mean weight change of − 2.3 kg (− 2.3, − 2.2), and (ii) change from baseline to completion, reporting a mean HbA1c change of − 2.0 mmol/mol (− 2.1, − 2.0) and a mean weight change of − 3.3 kg (− 3.4, − 3.2). In comparison, we found mean changes of − 1.6 mmol/mol (− 1.7, − 1.6) and − 3.2 kg (− 3.3, − 3.1) respectively among those retained to 6 months; − 2.1 mmol/mol (− 2.2, − 2.0) and − 3.6 kg (− 3.6, − 3.5) respectively among those retained to completion.

Our mean changes at completion, particularly regarding weight, are slightly larger than those in Valabhji et al., which could be explained by our stricter definition of completion: Valabhji et al. defined completion as attendance to at least 60% of sessions, whereas we additionally required participants to have attended the final intervention session or provide a final outcome measure [15]. Mean changes from baseline to the last available measure in Valabhji et al. are smaller than our 6 months and completion measures as the former will include participants who dropped out of the programme before 6 months. By only analysing participants who were retained to at least 6 months, we provide a more accurate estimation of the change in outcomes amongst participants. Since outcomes were not collected on participants who did not participate or who dropped out early on, we cannot estimate the effect of the intervention amongst all who initiated the programme, the Intention-to-Treat effect. Additionally, different baseline measurements of weight were also used, ours being at the initial assessment and Valabhji et al. used that at the first intervention session. The difference in choice of baseline may also in part, explain why our changes were larger as participants may have had reductions in weight or HbA1c between the initial assessment and the first session attended.

Our estimated mean weight loss at 6 months and completion are both larger than the pooled estimates in the systematic reviews by Ashra et al., Galaviz et al., Davies et al. and Dunkley et al. [8–11] They reported attending a diabetes prevention programme was associated with an overall mean weight change of − 2.46 kg [95% CI: − 2.99, − 1.94], − 2.5 kg [− 3.0, − 1.9], − 2.31 kg [− 2.87, − 1.76] and − 2.32 kg [− 2.92, − 1.72] respectively. Also, Ashra et al., Davies et al. and Dunkley et al. estimated the pooled change in HbA1c was −0.07% [95% CI: − 0.14, − 0.01] (~ − 0.7 mmol/mol), − 0.11% [− 0.19, − 0.03] (~ − 1.2 mmol/mol) and − 1.4 mmol/mol [− 2.4, − 0.5] respectively in those who received an intervention [8, 10, 11], all smaller than our estimated mean change in HbA1c. The differences in weight and HbA1c change may be partly due to differences in the follow-up times: the four systematic reviews included studies where the outcome was assessed longer than 12 months from baseline. Another difference is the study populations: studies in the systematic reviews had a broader inclusion criteria as participants could be at high risk of diabetes for any reason including age, family history and ethnicity, as well as weight and HbA1c.

Ashra et al. found the mean age of participants in the study was not associated with weight change but the studies with a higher proportion of males saw a higher incidence of diabetes and a smaller weight loss (although the latter was not statistically significant) [8]. Galaviz et al. and Dunkley et al. found the ethnicity mix in the study population had an impact on weight loss, with studies where participants were White or European had a larger weight loss than studies where participants were Hispanic or Asian, aligning with our findings [9, 11]. Ashra et al., Galaviz et al. and Dunkley et al. all found studies including a larger proportion of participants who were overweight or obese saw greater reductions in the onset of diabetes and larger weight loss. The suggestion that changes were greater in overweight and obese individuals may explain why we saw a greater mean weight loss than the pooled estimates from these systematic reviews as the majority (82.8%) of participants in our sample were classed as being overweight or obese at baseline. Dunkley et al. found significant FBG reductions were observed in studies where participants were over 50 years old, where participants were African American, in studies with at least 60% female participants, where at least 50% of participants had prediabetes and in studies where the BMI was < 30 [11].

### Strengths and limitations

This study had a large sample size and contained data from all participants who took part in the NHS DPP. A variety of patient information was collected enabling associations between these factors and outcomes to be assessed. Areas involved in the third wave of the rollout across England were not represented in the data, while some areas had more established services than others.

There could be variation in HbA1c measures between providers due to differences in devices and training. However, we have mitigated risk of bias as far as possible by using the change in HbA1c between initial assessment and 6 or 12 months, with each provider using the same device at all time points.

Analysis of outcomes were restricted to those retained to that time point, since outcomes for those lost to follow-up were thought likely to be missing not at random given observed data. Our results are therefore conditional on retention to at least 6 months; nothing can be inferred from these data about change in HbA1c or weight for those who left the programme before this stage. We expect average weight and HbA1c change would be lower if all individuals who started the programme had been analysed.

## Conclusions

On average, people who completed the programme saw a reduction in HbA1c of 2.1 mmol/mol (0.19%) and lost over 3.6 kg in weight. There was substantial variation in HbA1c change and smaller variation in weight loss between providers and across different sites. When different organisations provide a service, they may deliver it in different ways, which can lead to differences in health outcomes between providers. When implementing a Diabetes Prevention Programme, attention is needed to promote consistency, standardisation and learning across the providers and in different parts of the country.

These results were broadly similar regardless of patient characteristics, except reduction in HbA1c was slightly greater in non-smokers and weight loss was greater among people of White ethnicity and slightly greater in males. However it is important to note that the service did not measure HbA1c or weight change in people who left the programme by the time of measurement (and these were more likely to be Asian or Black people, younger people, those employed rather than retired and those who had a disability) [20].

## Supplementary Information

**Additional file 1.** Contains supplementary tables, additional information on coverage intervals used to illustrate variation in outcomes across sites, and additional information on missing data and multiple imputation.

**Additional file 2.** Contains information about how the sample was recruited, how representative the sample was of the target group, how the analysed sample differed from the recruited sample and how missing data were handled.

## Data Availability

The dataset analysed during the current study are not publicly available, nor available from the corresponding author on reasonable request. The data used is owned by NHS England and was shared with the research team as part of a Data Processing Agreement.
