# Supplementary material for: ‘Finishing the race’ – a cohort study of weight and blood glucose change among the first 36,000 patients in a large-scale diabetes prevention programme
Source: Int J Behav Nutr Phys Act. 2022 Jan 26;19:7. doi: 10.1186/s12966-022-01249-5 (PMC8793225; doi:10.1186/s12966-022-01249-5)
Supplement: Supplementary file 1 — Additional file 1. Contains supplementary tables, additional information on coverage intervals used to illustrate variation in outcomes across sites, and additional information on missing data and multiple imputation. [file 12966_2022_1249_MOESM1_ESM.docx]

**‘Finishing the race’ – A cohort study of weight and blood glucose change among the first 36,000 patients in a large-scale diabetes prevention programme.**

**Additional File 1**

**Table S1 Demographic summary and service characteristics for attenders referred between April 2016 and March 2018 - overall and by provider**

|  | **Attenders** | | | | | **Retained to 6m** | **Completed** |
| --- | --- | --- | --- | --- | --- | --- | --- |
|  | **Overall** | **Provider** | | | | **Overall** | **Overall** |
|  |  | **A** | **B** | **C** | **D** |  |  |
| **N (row %)** | 99,131 | 35,778  (36.1) | 12,930  (13.0) | 27,250  (27.5) | 23,173  (23.4) | 36,614 | 22,697 |
| **Sex**  Male  Female | 45,137  (45.7)  53,569  (54.2) | 15,860  (44.5)  19,740  (55.4) | 5,659  (43.9)  7,225  (56.1) | 12,401  (45.8)  14,649  (54.1) | 11,217  (48.4)  11,955  (51.6) | 16,479  (45.1)  20,018  (54.8) | 10,224  (45.2)  12,394  (54.8) |
| **Age (years)**  n  Median  (IQR) | 99,115  66  (17) | 35,778  66  (16) | 12,930  66  (17) | 27,234  65  (17) | 23,173  67  (16) | 36,614  68  (14) | 22,697  69  (12) |
| **Age group n (%)**  <40  40-49  50-59  60-69  70-79  80+ | 3,595  (3.6)  9,110  (9.2)  19,306  (19.5)  29,871  (30.1)  28,263  (28.5)  8,970  (9.1) | 1,146  (3.2)  3,023  (8.4)  7,071  (19.8)  11,054  (30.9)  10,129  (28.3)  3,355  (9.4) | 501  (3.9)  1,247  (9.6)  2,409  (18.6)  3,824  (29.6)  3,779  (29.2)  1,170  (9.1) | 1,187  (4.4)  2,834  (10.4)  5,573  (20.5)  7,923  (29.1)  7,401  (27.2)  2,316  (8.5) | 761  (3.3)  2,006  (8.7)  4,253  (18.4)  7,070  (30.5)  6,954  (30.0)  2,129  (9.2) | 574  (1.6)  1,994  (5.5)  5,873  (16.0)  12,528  (34.2)  12,482  (34.1)  3,163  (8.6) | 252  (1.1)  973  (4.3)  3,246  (14.3)  7,984  (35.2)  8,251  (36.4)  1,991  (8.8) |
| **Deprivation**  1 (most deprived)  2  3  4  5 (least deprived) | 18,990  (19.2)  19,532  (19.8)  19,901  (20.1)  19,782  (20.0)  20,625  (20.9) | 5,813  (16.2)  7,246  (20.3)  8,599  (24.0)  7,571  (21.2)  6,535  (18.3) | 4,073  (31.7)  2,793  (21.7)  2,465  (19.2)  1,732  (13.5)  1,783  (13.9) | 5,955  (22.0)  5,926  (21.9)  4,801  (17.8)  5,176  (19.1)  5,195  (19.2) | 3,149  (13.6)  3,567  (15.4)  4,036  (17.4)  5,303  (22.9)  7,112  (30.7) | 4,945  (13.5)  6,445  (17.6)  7,746  (21.2)  8,210  (22.5)  9,193  (25.2) | 2,755  (12.2)  4,080  (18.0)  5,047  (22.3)  5,192  (22.9)  5,578  (24.6) |
| **Ethnicity**^a^  White  Asian  Black  Mixed or other | 67,259  (76.1)  11,350  (12.9)  6,391  (7.2)  3,348  (3.8) | 26,621  (76.2)  4,402  (12.6)  2,423  (6.9)  1,507  (4.3) | 9,733  (77.5)  1,790  (14.2)  873  (7.0)  163  (1.3) | 14,450  (74.0)  2,216  (11.4)  2,131  (10.9)  731  (3.7) | 16,455  (77.2)  2,942  (13.8)  964  (4.5)  947  (4.4) | 27,499  (81.5)  3,099  (9.2)  2,014  (6.0)  1,133  (3.4) | 17,626  (83.9)  1,632  (7.8)  1,116  (5.3)  632  (3.0) |
| **Employment**  Employed  Retired  Other | 23,740  (31.8)  43,007  (57.7)  7,811  (10.5) | 11,551  (33.2)  19,483  (56.1)  3,702  (10.7) | 3,594  (30.5)  6,707  (56.9)  1,487  (12.6) | 3,490  (30.0)  7,210  (61.9)  944  (8.1) | 5,105  (31.2)  9,607  (58.6)  1,678  (10.2) | 7,285  (25.8)  18,791  (66.5)  2,183  (7.7) | 4,227  (23.5)  12,570  (69.8)  1,219  (6.8) |
| **Disability**  No  Yes | 68,927  (82.6)  14,533  (17.4) | 28,695  (82.5)  6,069  (17.5) | 8,231  (75.2)  2,718  (24.8) | 14,579  (89.6)  1,696  (10.4) | 17,422  (81.1)  4,050  (18.9) | 27,107  (84.5)  4,982  (15.5) | 16,917  (85.1)  2,957  (14.9) |
| **Smoking**  Smoker  Ex-smoker  Non-smoker | 5,794  (8.2)  1,684  (2.4)  63,327  (89.4) | 3,080  (9.2)  2  (0.0)  30,513  (90.8) | 983  (8.7)  1,003  (8.9)  9,320  (82.4) | 651  (5.6)  314  (2.7)  10,592  (91.7) | 1,080  (7.5)  365  (2.5)  12,902  (89.9) | 1,381  (5.0)  574  (2.1)  26,619  (92.9) | 801  (4.6)  326  (1.9)  16,374  (93.6) |
| **WEMWBS score**  N Median (IQR) | 46,718  54  (13) | 17,839  54  (12) | 7,131  54  (14) | 13,614  54  (15) | 8,134  52  (14) | 18,569  54  (12) | 11,936  55  (12) |
| **EQ-VAS**  N Median (IQR) | 30,518  80  (25) | 11,136  75  (25) | 5,761  80  (30) | 7,221  80  (30) | 6,400  80  (20) | 10,894  80  (20) | 6,675  80  (20) |
| **Referral source**  Consultation (GP/ health check)  Letter (self-referral after advice) | 63,201  (63.8)  35,906  (36.2) | 20,689  (57.8)  15,085  (42.2) | 12,046  (93.2)  865  (6.7) | 7,293  (26.8)  19,956  (73.2) | 23,173  (100.0)  0  (0.0) | 23,901  (65.3)  12,705  (34.7) | 13,665  (60.2)  9,027  (39.8) |
| **Out-of-hours provision**  None  Some | 83,754  (84.5)  15,371  (15.5) | 32,001  (89.5)  3,773  (10.5) | 12,056  (93.2)  874  (6.8) | 24,733  (90.8)  2,517  (9.2) | 14,964  (64.6)  8,207  (35.4) | 29,699  (81.1)  6,910  (18.9) | 19,230  (84.7)  3,467  (15.3) |

^a^ ‘Asian’ comprises those reporting Indian, Pakistani, Bangladeshi, Chinese or ‘other Asian’ ethnicity; ‘Black’ comprises those reporting Caribbean, African or ‘other Black’ ethnicity; ‘Mixed’ comprises people with a Mixed ethnic background and ‘other’ comprises those reporting any other ethnicity.

**Table S2 Health measures at initial assessment, 6 months and completion**

|  | **Attenders**  **(n=99,131)** | **Retained to 6m**  **(n=36,614)** | | **Completed**  **(n=22,697)** | |
| --- | --- | --- | --- | --- | --- |
|  | **IA** | **IA** | **6 months** | **IA** | **final** |
| **HbA1c (mmol/mol)** |  |  |  |  |  |
| n  Median  (IQR) | 67,697  41  (5) | 24,568  41  (5) | 29,086  39  (5) | 16,164  42  (5) | 20,272  39  (4) |
| **HbA1c (%)** |  |  |  |  |  |
| n  Median  (IQR) | 67,697  5.9  (0.5) | 24,568  5.9  (0.5) | 29,086  5.7  (0.5) | 16,164  6.0  (0.4) | 20,272  5.7  (0.4) |
| **FPG (mmol/l)** |  |  |  |  |  |
| n  Median  (IQR) | 49  6.0  (0.6) | 15  6.3  (0.8) | 151  5.8  (0.3) | 9  6.2  (0.6) | 41  5.7  (0.4) |
| **Blood glucose category**^a^ **n (%)** |  |  |  |  |  |
| Normal | 35,311  (52.1) | 12,397  (50.4) | 21,059  (72.4) | 7,937  (49.1) | 15,642  (77.2) |
| NDH | 28,684  (42.3) | 11,389  (46.3) | 7,371  (25.3) | 7,747  (47.9) | 4,298  (21.2) |
| T2DM | 3,749  (5.5) | 797  (3.2) | 660  (2.3) | 489  (3.0) | 333  (1.6) |
| **Weight (overall)** |  |  |  |  |  |
| n  Median  (IQR) | 89,761  82  (24.1) | 34,153  81.4  (23.5) | 26,832  77.6  (22.6) | 21,113  80.8  (23.0) | 21,291  77.0  (22.5) |
| **BMI category**^b^ **n (%)** |  |  |  |  |  |
| Underweight or healthy weight | 15,329  (17.2) | 5,866  (17.3) | 6,897  (26.1) | 3,809  (18.2) | 5,819  (27.8) |
| Overweight | 32,884  (37.0) | 13,108  (38.7) | 10,397  (39.4) | 8,296  (39.5) | 8,291  (39.6) |
| Obese | 40,713  (45.8) | 14,887  (44.0) | 9,091  (34.5) | 8,872  (42.3) | 6,808  (32.6) |

Abbreviations: IA – initial assessment, NDH – non-diabetic hyperglycaemia, T2D – type 2 diabetes

^a^HbA1c and FPG groups combined; ‘Normal’ range: HbA1c<42mmol/mol or FPG<5.5mmol/l; ‘NDH’ range: HbA1c 42-47mmol/mol or FPG 5.5-6.9mmol/l inclusive; ‘T2DM’ range: HbA1c>47mmol/mol or FPG>6.9mmol/l

^b^ ‘Underweight’: <18.5kg/m^2^; ‘healthy weight’: 18.5-24.9kg/m^2^; ‘overweight’: 25-29.9kg/m^2^ and ‘obese’: 30kg/m^2^ or more. See previous note on interpretation.

**Table S3 Changes in HbA1c and weight from initial assessment to six months (n=36607) and completion (m=22697) by patient and service characteristics**

|  | **IA-6m** | **IA-6m** | **IA-final** | **IA-final** |
| --- | --- | --- | --- | --- |
|  | **Change in HbA1c (mmol/mol)** | **Change in weight (kg)** | **Change in HbA1c (mmol/mol)** | **Change in weight (kg)** |
| n  Mean (SD) | 20,682  -1.7 (4.3) | 24,949  -3.2 (4.4) | 15,017  -2.3 (4.3) | 19,794  -3.6 (4.8) |
| **Sex** |  |  |  |  |
| Male | 9,212  -1.9 (4.4) | 11,096  -3.6 (4.7) | 6,714  -2.5 (4.3) | 8,888  -3.9 (5.1) |
| Female | 11,389  -1.6 (4.2) | 13,764  -2.9 (4.1) | 8,242  -2.2 (4.3) | 10,833  -3.3 (4.6) |
| **Age (years)** |  |  |  |  |
| <40 | 289  -1.6 (4.7) | 345  -2.4 (5.3) | 165  -2.0 (5.1) | 216  -2.3 (6.2) |
| 40-49 | 1,044  -1.5 (5.3) | 1,209  -2.6 (5.1) | 637  -1.9 (4.7) | 807  -2.9 (6.1) |
| 50-59 | 3,197  -1.7 (4.5) | 3,811  -2.9 (4.9) | 2,152  -2.2 (4.7) | 2,806  -3.1 (5.3) |
| 60-69 | 7,267  -1.8 (4.2) | 8,717  -3.4 (4.6) | 5,336  -2.3 (4.3) | 6,995  -3.7 (5.0) |
| 70-79 | 7,099  -1.8 (4.1) | 8,745  -3.4 (4.0) | 5,410  -2.4 (4.1) | 7,214  -3.8 (4.4) |
| 80+ | 1,777  -1.6 (4.0) | 2,122  -3.0 (3.7) | 1,317  -2.2 (4.1) | 1,756  -3.4 (3.7) |
| **Deprivation** |  |  |  |  |
| 1 (most deprived) | 2,658  -1.4 (4.5) | 3,284  -2.8 (4.6) | 1,794  -2.1 (4.6) | 2,390  -3.1 (5.0) |
| 2 | 3,727  -1.8 (4.2) | 4,440  -3.2 (4.5) | 2,781  -2.3 (4.7) | 3,563  -3.5 (5.1) |
| 3 | 4,591  -1.7 (4.2) | 5,556  -3.2 (4.3) | 3,497  -2.3 (4.0) | 4,496  -3.5 (4.8) |
| 4 | 4,638  -1.8 (4.3) | 5,609  -3.4 (4.4) | 3,363  -2.3 (4.2) | 4,524  -3.8 (4.7) |
| 5 (least deprived) | 5,027  -1.9 (4.3) | 6,016  -3.3 (4.4) | 3,555  -2.4 (4.3) | 4,790  -3.7 (4.7) |
| **Ethnicity** |  |  |  |  |
| White | 15,685  -1.7 (4.1) | 19,318  -3.4 (4.5) | 11,620  -2.3 (4.1) | 15,593  -3.8 (4.9) |
| Asian | 1,662  -1.5 (4.5) | 1,970  -1.9 (3.7) | 1,133  -2.0 (4.2) | 1,466  -2.2 (4.0) |
| Black | 1,107  -1.4 (4.7) | 1,290  -2.3 (4.0) | 758  -1.7 (5.8) | 956  -2.4 (4.7) |
| Mixed or other | 575  -1.9 (4.1) | 713  -2.8 (4.1) | 391  -2.0 (4.3) | 547  -3.2 (4.5) |
| **Employment** |  |  |  |  |
| Employed | 4,107  -1.9 (4.2) | 5,089  -2.9 (4.6) | 2,905  -2.2 (4.3) | 3,806  -3.1 (5.0) |
| Retired | 10,667  -1.8 (3.9) | 13,730  -3.4 (4.2) | 8,351  -2.3 (3.9) | 11,364  -3.8 (4.6) |
| Other | 1,144  -1.7 (4.1) | 1,463  -2.6 (4.7) | 813  -2.3 (4.2) | 1,083  -2.9 (5.3) |
| **Disability** |  |  |  |  |
| No | 15,399  -1.8 (4.1) | 18,856  -3.2 (4.3) | 11,206  -2.3 (4.2) | 15,009  -3.5 (4.8) |
| Yes | 2,571  -1.5 (3.9) | 3,352  -3.1 (4.6) | 1,833  -2.2 (4.0) | 2,664  -3.6 (5.2) |
| **Smoking** |  |  |  |  |
| Smoker | 735  -1.2 (4.0) | 965  -2.9 (4.6) | 525  -1.8 (4.0) | 733  -3.1 (5.4) |
| Ex-smoker | 280  -1.8 (4.1) | 411  -3.4 (4.6) | 170  -2.0 (3.8) | 303  -3.7 (4.8) |
| Non-smoker | 14,921  -1.7 (4.0) | 18,533  -3.3 (4.4) | 11,020  -2.3 (4.1) | 14,855  -3.6 (4.8) |
| **Blood glucose category at IA** |  |  |  |  |
| Normal | 10,413  -0.1 (3.7) | 9,097  -2.8 (4.3) | 7,232  -0.5 (3.8) | 6,984  -3.0 (4.6) |
| NDH | 9,598  -3.2 (3.9) | 8,584  -3.4 (4.5) | 7,331  -3.7 (3.7) | 6,965  -3.8 (4.9) |
| T2DM | 671  -6.7 (6.1) | 545  -3.9 (5.1) | 454  -7.6 (6.9) | 410  -4.3 (5.8) |
| **BMI at IA** |  |  |  |  |
| Underweight or healthy weight | 3,423  -1.5 (4.1) | 4,465  -1.8 (3.3) | 2,578  -2.1 (4.1) | 3,601  -2.0 (3.3) |
| Overweight | 7,384  -1.7 (4.0) | 9,613  -3.0 (3.7) | 5,480  -2.2 (4.5) | 7,804  -3.3 (4.0) |
| Obese | 8,275  -1.8 (4.3) | 10,723  -4.0 (5.2) | 5,724  -2.4 (4.5) | 8,262  -4.5 (5.8) |
| **Provider** |  |  |  |  |
| A | 10,065  -2.0 (3.5) | 13,016  -3.3 (4.3) | 8,270  -2.5 (3.6) | 10,839  -3.6 (4.8) |
| B | 2,143  -0.9 (3.2) | 3,238  -2.8 (4.5) | 1,518  -0.9 (3.1) | 2,291  -3.0 (4.8) |
| C | 5,783  -2.0 (5.5) | 4,953  -3.2 (4.8) | 4,353  -2.7 (5.4) | 3,901  -3.6 (5.0) |
| D | 2,691  -0.8 (4.5) | 3,742  -3.2 (4.1) | 876  -1.0 (4.8) | 2,763  -3.7 (4.7) |
| **Referral source** |  |  |  |  |
| Consultation (GP/ health check) | 11,417  -1.5 (4.0) | 15,422  -3.3 (4.3) | 7,722  -2.0 (3.9) | 12,044  -3.6 (4.8) |
| Letter (self-referral after advice) | 9,259  -2.0 (4.6) | 9,520  -3.2 (4.5) | 7,291  -2.6 (4.7) | 7,745  -3.5 (4.8) |
| **Out-of-hours provision** |  |  |  |  |
| None | 17,574  -1.8 (4.2) | 21,131  -3.3 (4.4) | 13,140  -2.3 (4.3) | 16,991  -3.6 (4.8) |
| Some | 3,106  -1.4 (4.7) | 3,818  -2.8 (4.2) | 1,877  -2.0 (4.4) | 2,803  -3.0 (4.7) |

Abbreviations: IA – initial assessment, NDH – non-diabetic hyperglycaemia, T2D – type 2 diabetes

**Table S4 Associations of patient and service characteristics with change in HbA1c and weight at completion**

|  | **Change in HbA1c IA-final** | | | **Change in weight (kg) IA-final** | | |
| --- | --- | --- | --- | --- | --- | --- |
|  | **Coef** | **SE** | **95% CI** | **Coef** | **SE** | **95% CI** |
| **Sex**^a^ | | | | | | |
| Female | 0.27 | 0.08 | 0.12, 0.42 | 0.58 | 0.08 | 0.42, 0.75 |
| Male | 0 (ref) |  |  | 0 (ref) |  |  |
| **Age per 5 years**^a^ | 0.002 | 0.02 | -0.05, 0.05 | -0.02 | 0.02 | -0.07, 0.02 |
| **Ethnicity**^ab^ | | | | | | |
| Asian | 0.23 | 0.17 | -0.10, 0.56 | 1.47 | 0.16 | 1.16, 1.79 |
| Black | 0.48 | 0.23 | 0.03, 0.93 | 1.07 | 0.18 | 0.71, 1.43 |
| Other | 0.41 | 0.26 | -0.11, 0.93 | 0.44 | 0.20 | 0.05, 0.83 |
| White | 0 (ref) |  |  | 0 (ref) |  |  |
| **Deprivation**^cd^ |  |  |  |  |  |  |
| 1 (most deprived) | 0.16 | 0.14 | -0.12, 0.44 | 0.08 | 0.13 | -0.16, 0.33 |
| 2 | -0.09 | 0.12 | -0.32, 0.14 | -0.20 | 0.11 | -0.41, 0.01 |
| 3 | -0.06 | 0.11 | -0.28, 0.16 | -0.11 | 0.10 | -0.31, 0.09 |
| 4 | 0.07 | 0.10 | -0.13, 0.27 | -0.22 | 0.10 | -0.41,  -0.02 |
| 5 (least deprived) | 0 (ref) |  |  | 0 (ref) |  |  |
| **Disability**^cd^ |  |  |  |  |  |  |
| Yes | 0.02 | 0.11 | -0.20, 0.24 | 0.01 | 0.10 | -0.19, 0.22 |
| No | 0 (ref) |  |  | 0 (ref) |  |  |
| **Employment**^cd^ |  |  |  |  |  |  |
| Employed | -0.03 | 0.13 | -0.28, 0.23 | 0.50 | 0.11 | 0.28, 0.72 |
| Retired | 0 (ref) |  |  | 0 (ref) |  |  |
| Other | -0.24 | 0.17 | -0.58, 0.10 | 0.41 | 0.18 | 0.06, 0.77 |
| **Smoking**^cd^ | | | | | | |
| Ex- or current smoker | 0.56 | 0.20 | 0.16, 0.97 | 0.44 | 0.19 | 0.07, 0.82 |
| Non-smoker | 0 (ref) |  |  | 0 (ref) |  |  |
| **IA HbA1c (per mmol/mol)**^de^ | n/a | n/a | n/a | -0.10 | 0.01 | -0.13, -0.08 |
| **IA weight (per 5kg)**^de^ | -0.05 | 0.01 | -0.07, -0.02 |  |  |  |
| **IA WEMWBS (per 5 points)**^df^ | -0.01 | 0.03 | -0.06, 0.04 | -0.05 | 0.03 | -0.10, 0.01 |
| **IA EQVAS (per 5 points)**^df^ | -0.01 | 0.02 | -0.05, 0.04 | 0.01 | 0.02 | -0.03, 0.05 |
| **Provider**^dg^ |  |  |  |  |  |  |
| A | -1.86 | 0.26 | -2.36, -1.35 | -0.56 | 0.18 | -0.92, -0.20 |
| B | 0 (ref) |  |  | 0 (ref) |  |  |
| C | -2.25 | 0.28 | -2.80, -1.70 | -0.78 | 0.20 | -1.17, -0.39 |
| D | -0.20 | 0.33 | -0.85, 0.46 | -0.70 | 0.21 | -1.11, -0.28 |
| **Referral source**^dg^ |  |  |  |  |  |  |
| Letter | 0.04 | 0.10 | -0.15, 0.23 | 0.21 | 0.10 | 0.02, 0.40 |
| Consultation | 0 (ref) |  |  | 0 (ref) |  |  |
| **Out-of-hours provision**^dg^ |  |  |  |  |  |  |
| Some | -0.004 | 0.11 | -0.22, 0.21 | 0.36 | 0.10 | 0.16, 0.57 |
| None | 0 (ref) |  |  | 0 (ref) |  |  |
| ${\hat{\boldsymbol{\sigma}}}_{\boldsymbol{STP}}^{\boldsymbol{2}}\boldsymbol{+}{\hat{\boldsymbol{\sigma}}}_{\boldsymbol{CCG}}^{\boldsymbol{2}}$ | 0.51 | | | 0.17 | | |
| **ICC (CCG level)** | 0.03 | | | 0.01 | | |

For categorical variables, ‘coef’ represents the adjusted difference in mean change in relation to the reference category. For numerical variables, ‘coef’ represents the adjusted difference in mean change between consecutive values. Change is calculated as the final value minus the baseline value

^a^ Associations of sex, age and ethnicity with each outcome were mutually adjusted and marginal over site (no random terms).

^b^ ‘Asian’ comprises those reporting Indian, Pakistani, Bangladeshi, Chinese or ‘other Asian’ ethnicity; ‘Black’ comprises those reporting Caribbean, African or ‘other Black’ ethnicity; ‘Mixed’ comprises people with a Mixed ethnic background and ‘other’ comprises those reporting any other ethnicity.

^c^ Associations of disability, employment, deprivation and smoking with each outcome were mutually adjusted and adjusted for sex, age and ethnicity.

^d^ Random intercept terms for STP & CCG included; associations are conditional, representing the estimated individual-level mean association after random variation between sites has been accounted for.

^e^ Association of initial weight with change in HbA1c, and of initial HbA1c with change in weight, was adjusted for sex, age, ethnicity, employment, disability, deprivation and smoking.

^f^ Association of WEMWBS score or EQVAS score with change in HbA1c [weight] was adjusted for sex, age, ethnicity, employment, disability, deprivation, smoking and initial weight [HbA1c].

^g^ Associations of service features with change in HbA1c [weight] were mutually adjusted and adjusted for sex, age, ethnicity, deprivation, smoking, initial weight [HbA1c] and service maturity.

**Detail of coverage intervals used to illustrate variation in outcomes across sites**

Model variance estimates for random intercept terms (for CCG nested in STP) were examined in order to illustrate the variation in predicted outcomes due to variation between sites, after adjustment for patient and service characteristics. STP and CCG effects were assumed to be independently Normally distributed with mean 0 and variance $\hat{\sigma}_{\mathrm{STP}}^{2}$ and $\hat{\sigma}_{\mathrm{CCG}}^{2}$ respectively; it is therefore expected that around 95% of sites will have combined site effects ($u_{\mathrm{STP}}+u_{\mathrm{CCG}}$) within $\pm1.96\sqrt{\hat{\sigma}_{\mathrm{STP}}^{2}+\hat{\sigma}_{\mathrm{CCG}}^{2}}$ of 0. Together with the model assumptions, these were used to calculate coverage intervals giving the predicted mean value of each outcome across the middle 95% of sites, for a reference group with ‘typical’ values for individual and service characteristics.(22) Typical characteristics were chosen to be the modal value for sex, disability, employment, smoking, referral source and provider, and values close to the median for other covariates, representing a White, 65 year old, retired, non-smoking woman in the middle deprivation group, reporting no disability, with HbA1c of 41mmol/mol, weight 81kg, referred via consultation to a six month old service with provider A.

**Detail of missing data and multiple imputation**

Multiple imputation was used to reduce bias due to missingness among covariate values of up to 44%, see Table S5 for details. Fully conditional specification (FCS) was used due to the large number of variables of mixed continuous and categorical types to be used.(23, 24) Planned analyses and therefore imputation were restricted to those retained to at least six months; imputation was performed separately for completers and non-completers since imputation model coefficients may plausibly have differed between these groups. Outcomes were included in imputation models and imputed where due for collection, though final outcomes were not imputed for non-completers since these were missing by design. Imputed WEMWBS scores were set to missing for those where EQ-VAS scores were expected and vice-versa, so that participants were only included in analysis of the scale used at the time of their referral. This approach ensured that for each analysis, all covariates, relevant outcomes and auxiliary variables were included in the FCS for the corresponding subsample.(25) Forty imputations were performed to give acceptably low Monte Carlo error, using a burn-in of 50, chosen after examination of trace plots to check for convergence.(23)

After small groups had been excluded (those with sex missing (n=102) or reported as ‘other’ (n=15), those with deprivation missing (n=75), those referred via methods other than consultation or letter (n=8), those transferred to wave 3 or 4 services (n=37); n=229 in total giving a reduced cohort of n=36,379 of which 22,554 completed), complete covariates: sex, age, deprivation, provider, referral source, out-of-hours delivery, service maturity (months since establishment to referral) and STP (site); incomplete covariates: ethnicity, occupation, disability, smoking, HbA1c at initial assessment , weight at initial assessment, WEMWBS score at initial assessment, EQ-VAS score at initial assessment; incomplete outcomes: change in HbA1c to 6m, change in weight to 6m; and auxiliary variables height (incomplete), referral HbA1c (incomplete), wave (local phase of DPP implementation, complete) and neighbourhood proportion of Black and minority ethnic (BAME) residents obtained from census data 2011 (complete) were included in the FCS for both retention groups. Incomplete outcomes change in HbA1c and weight to completion were added to the FCS for completers.

Multinomial logistic imputation models were used for categorical variables, with augmented regression to overcome perfect prediction of categorical variables (ethnicity, employment and smoking).(26) Predictive mean matching was used for all baseline measures and change outcomes due to skewed distributions among observed data. Linear regression was used for height. Clustering by site was accounted for by including STP as a covariate in the imputation models (subgroups defined by CCG were too small to include CCG as a covariate).

**Table S5 Variables used in multiple imputation models (after small groups removed, n=229)**

|  | **% missing** | |
| --- | --- | --- |
|  | **Retained to 6 months**  **n=36,379** | **Completed**  **n=22,554** |
| **Substantive covariates** |  |  |
| Sex | Complete | |
| Age | Complete | |
| Deprivation | Complete | |
| Provider | Complete | |
| Referral source | Complete | |
| Out-of-hours provision | Complete | |
| Service maturity | Complete | |
| STP | Complete | |
| Ethnicity | 8 | 7 |
| Employment | 23 | 21 |
| Disability | 12 | 12 |
| Smoking | 25 | 23 |
| HbA1c at IA | 33 | 29 |
| Weight at IA | 7 | 7 |
| WEMWBS at IA (where due) | 15 | 15 |
| EQ-VAS at IA (where due) | 44 | 42 |
|  |  |  |
| **Outcomes** |  |  |
| Change in HbA1c at 6m | 43 | 36 |
| Change in weight at 6m | 32 | 20 |
| Change in HbA1c at completion | n/a | 34 |
| Change in weight at completion | n/a | 13 |
| Change in WEMWBS at completion (where due) | n/a | 53 |
| Change in EQ-VAS at completion (where due) | n/a | 52 |
|  |  |  |
| **Auxiliary variables** |  |  |
| log(neighbourhood proportion BAME residents) | Complete | |
| Height | 2 | 2 |
| HbA1c at referral | 11 | 9 |
