# Supplementary material for: ‘Finishing the race’ – a cohort study of weight and blood glucose change among the first 36,000 patients in a large-scale diabetes prevention programme
Source: Int J Behav Nutr Phys Act. 2022 Jan 26;19:7. doi: 10.1186/s12966-022-01249-5 (PMC8793225; doi:10.1186/s12966-022-01249-5)
Supplement: Supplementary file 2 — Additional file 2. Contains information about how the sample was recruited, how representative the sample was of the target group, how the analysed sample differed from the recruited sample and how missing data were handled. [file 12966_2022_1249_MOESM2_ESM.docx]

**‘Finishing the race’ – A cohort study of weight and blood glucose change among the first 36,000 patients in a large-scale diabetes prevention programme.**

**Additional File 2**

***How the sample was recruited***

The sample in the dataset used for the analysis comprises participation and outcome data for all individuals referred to the NHS Diabetes Prevention Programme between the start of the programme in April 2016 and September 2019. The target population for the programme was adults aged 18 and over with non-diabetic hyperglycaemia (NDH), defined as HbA1c of 42–47mmol/mol (6·0–6·4%) or fasting plasma glucose level (FPG) of 5·5–6·9mmol/l. Eligible participants were referred to the programme through their GP via one of two main routes: i) referral by a primary care professional following a consultation, or ii) self-referral following receipt of a letter from their GP, informing them of their high risk of type 2 diabetes (based on their medical records) and encouraging them to participate.

***How representative the sample was of the target group***

Data on individuals who declined the offer of joining the programme were not collected.

This paper focusses on the outcomes of participants of programme, specifically those who were still attending sessions 6 months after the initial assessment and those who completed the programme. The sample is therefore fully representative of all participants on the programme. However, as outcome data on individuals who were referred but did not subsequently participate were not collected, our analysis was not able to estimate the average outcome of all individuals referred to the programme.

***How the analysed sample differed from the recruited sample***

As mentioned, only individuals who were still participating in the programme at six months and those who completed the programmed were analysed. An investigation of factors associated with participation in the programme has previously been undertaken and published by our research team.(1)

Table 1 and Table S1 summarise the baseline characteristics of those who (1) started the programme by attending an initial assessment, (2) those who were retained at 6 months and (3) those who completed the programme. The median age of those retained to 6 months and completion was slightly higher than that of all who attended an initial assessment. The proportion of individuals of White ethnicity, retired individuals, those with no disability and non-smokers was higher amongst those retained to 6 months and completion, compared to all those who attended an initial assessment.

***How missing data were handled***

Multiple imputation was used to reduce bias due to missingness of outcomes and demographic data. Missing outcome data at six months was imputed for individuals who were classed as still participating in the programme at this time point (those with any final measure recorded or a period of attendance of at least 180 days from the first attended intervention session) and missing outcome data at completion was imputed for individuals who had completed the programme (those who had attended at least 60% of all intervention sessions offered, including the final sessions). Fully conditional specification (FCS) was used due to the large number of variables of mixed continuous and categorical types to be used. Further detail is given in Additional File 1.

1. Howarth E, Bower PJ, Kontopantelis E, Soiland-Reyes C, Meacock R, Whittaker W, et al. 'Going the distance': an independent cohort study of engagement and dropout among the first 100 000 referrals into a large-scale diabetes prevention program. BMJ Open Diabetes Res Care. 2020;8(2).
